# Hepatoprotective activity of *Mammea africana* ethanol stem bark extract 

**Published:** 2016

**Authors:** Jude Efiom Okokon, Michael Burata Bawo, Herbert Orji Mbagwu

**Affiliations:** *Department of Pharmacology and Toxicology Faculty of Pharmacy, University of Uyo, Uyo, Nigeria *

**Keywords:** *Medicinal plant*, *Mammea Africana*, *Hepatoprotective*, *Antioxidant*

## Abstract

**Objective:**

The stem bark of* Mammea africana* Sabine (Guttiferae), (*M. africana*) a common plant that has been traditionally used to treat various diseases and ailments was evaluated for hepatoprotective potentials against paracetamol-induced liver injury in rats.

**Materials and Methods:**

The hepatoprotective effect of the stem bark extract (30-90 mg/kg) was evaluated by the assay of liver function parameters, namely total and direct bilirubin, serum protein and albumin, total cholesterol, alanine aminotransaminase (ALT), aspartate aminotransaminase (AST), and alkaline phosphatase activities (ALP), antioxidant enzymes: superoxide dismutase (SOD), catalase (CAT), glutathione peroxidase (GPx), reduced glutathione (GSH) and histopathological study of the liver.

**Results:**

Administration of the stem bark extract caused a significant (p<0.05 – 0.001) dose-dependent reduction of high levels of liver enzymes (ALT, AST and ALP), total cholesterol, direct and total bilirubin as well as elevation of serum levels of total protein, albumin and antioxidant enzymes (SOD, CAT, GPx and GSH). Histology of the liver sections of extract and silymarin-treated animals showed reductions in the pathological features compared to the paracetamol-treated animals. The chemical pathological changes were consistent with histopathological observations suggesting marked hepatoprotective effect of the stem bark extract of *M. africana. *

**Conclusion:**

The results show that the stem bark extract of *M. africana* has hepatoprotective potential which may be due to its antioxidant activity.

## Introduction

*Mammea africana* Sabine (Guttiferae) (syn. *Ochrocarpus africana *Oliv.) (*M. africana*) is a large forest tree of 50 to 100 feet high with bark often yellow with pale scales and resinous yellow sap (Hutchison and Daziel, 1958 ▶). The plant is widely distributed in tropical Africa. The stembark of the plant is traditionally used by the Ibibios, of Niger Delta region of Nigeria, in the treatment of malaria related fever, diabetes, microbial infections and mental disorders. The stembark is also traditionally used to treat stomach pains, rheumatism pains, scabies, cough and hypertension (Raponda-Walter and Sillans, 1961 ▶; Adjanohoun et al., 1996 ▶). The stembark extract has been reported to possess cytotoxic activity, *in vitro* (Chapuis et al., 1988 ▶;Okokon et al., 2012 ▶). Ouahouo et al., (2004) ▶ reported cytotoxic coumarins with anti-microbial activity against *Staphylococcus aureus* from the plant stembark. The stembark has been reported to have anti-plasmodial (Okokon et al.,2006 ▶), cardioprotective (Okokon and Antia,2007 ▶), anti-diabetic , hypolipidaemic (Okokon et al.,2007 ▶), vasorelaxant (Dongmo et al.,2007 ▶), anti-hypertensive (Nguelefack-Mbuyo et al., 2008 ▶), anti-inflammatory, analgesic (Okokon et al.,2009 ▶), antioxidant (Nguelefack-Mbuyo et al., 2010 ▶), anti-diarrheal, anti-ulcer (Okokon et al., 2010 ▶), immunomodulatory, anti-lesihmanial (Okokon et al., 2012 ▶), depressant and anti-convulsant (Okokon and Davis, 2014 ▶) as well as nephroprotective (Okokon and Bawo, 2014 ▶) activities. The stembark has been reported to have 5,-7-dihydroxy-8-(12-methyl-butryl) – 4 –N -pentylcoumarins (Carpenter et al., 1970 ▶, 1971; Crichton and Waterman, 1978 ▶), 4-phenyl and 4-alkylcoumarins (Games, 1972 ▶), mesuxanthone B (Carpenter et al., 1971 ▶). Alkaloids have been reported to be absent in the entire plant parts (Gartlands et al., 1980 ▶). 

We, therefore, report in this study the hepatoprotective activity of the stembark extract of *M. africana* from Nigeria. 

## Materials and methods

**Plant collection**

The plant material *M. africana *(stembark) was collected in April 2013 from a forest in Uruan area, Akwa Ibom State, Nigeria. The plant was identified and authenticated by Dr. Magaret Bassey, Department of Botany and Ecological Studies, University of Uyo, Uyo, Nigeria. Herbarium specimen was deposited at the hebarium of Faculty of Pharmacy with voucher no. FPHUU 381.

**Extraction**

The pieces of the stembark were washed and shade-dried for two weeks. The dried plants’ materials were further chopped into smaller pieces and grounded to powder. The powdered material was soaked in 70% ethanol for 72 hr. The liquid filtrates were concentrated and evaporated to dryness *in vacuo* at 40˚C using rotary evaporator (Okokon et al., 2006 ▶). 

**Animals**

Swiss albino rats of either sex (190 – 220 g) used for these experiments were obtained from University of Uyo animal house. The animals were housed in standard cages and maintained on a standard pelleted feed (Guinea feed) and water was *ad libitum*. Permission and approval for animal studies were obtained from College of Health Sciences Animal Ethics committee, University of Uyo (UU/CHSAE/14/012).

**Animal treatment**

A total of 36 rats of both sexes were weighed and divided into six groups of 6 animals each and treated as follows: Group A consisted of normal animals that were administered with distilled water (0.2 ml/kg), Group B was administered with distilled water 0.2 ml/kg, while groups C, D and E were administered with p.o 30, 60 and 90 mg/kg/day of *M. africana *stembark extract (MASBEX), for 8 days, respectively. Group F was treated with silymarin (100 mg/kg) (standard/reference drug representing positive control) for the same period of time. Paracetamol, 2 g/kg, was administered to groups B - F on the eighth day. Twenty-four hours after paracetamol administration, the animals were sacrificed under light diethyl ether vapor. Blood were collected by cardiac puncture and used immediately.

**Hematological study**

Animals were sacrificed under diethyl ether anaesthesia, blood samples were collected from each rat by cardiac puncture using 21 gauge (21 G) needles mounted on a 5 ml syringe into ethylene diamine tetra-acetic acid (EDTA) - coated sample bottles for analysis. Hematological parameters such as full blood count (FBC), hemoglobin, (Hb), packed cell volume (PCV), platelet concentration (PLC) and total and differential white blood cell count (WBC) were recorded. These parameters were analyzed using automatic hematological system (Sysmex Hematology – Coagulation system, Model MO-1000 I, Trans Asia, Japan).

**Evaluation of the** **protective** **effect of** **the** **extract** **against** **paracetamol** **-induced** **liver** **injury** **regarding biochemical parameters** **and h** **istology of** **liver** **of rats**

 Serum was separated from the blood samples and the sera were stored at -20^o^C until used for biochemical determinations such as total protein, albumin, aspartate aminotransferase (AST), alanine aminotransferase (ALT), alkaline phosphatase(ALP), total cholesterol, total and direct bilirubin. The determinations were done spectrophotometrically using Randox analytical kits according to standard procedures of manufacturer’s protocols (Reitman and Frankel, 1957 ▶). The livers of the animals were surgically removed, weighed and a part of each was fixed in 10% formaldehyde for histological processes, and the rest was washed with ice cold 0.9% NaCl and homogenates were made at a ratio of 1 g of wet tissue to 9 ml of 1.25% KCl using motor driven Teflon-pestle. The homogenates were centrifuged at 7000 rpm for 10 min at 4˚C and the supernatants were used for the assays of superoxide dismutase (SOD) (Marklund et al., 1974 ▶), catalase (CAT) (Sinha, 1972 ▶), glutathione peroxidase (GPX) (Lawrence and Burk, 1976 ▶), and reduced glutathione (GSH) (Ellman, 1959 ▶).

**Statistical analysis**

 Data obtained from this work were analyzed statistically using Student’s t-test and ANOVA (One - way) followed by a post test (Tukey-Kramer multiple comparison test). Differences between means were considered significant at p < 0.05.

## Results

**Effect of ethanolic extract of** ***M. africana*** **stembark on the hematological parameters of rats with paracetamol-induced hepatotoxicity**

Administration of paracetamol (2 g/kg) to rats did not affect (p<0.05) RBC and WBC counts as well as PCV percentage and hemoglobin concentration significantly (Table 2). However, there were significant (p<0.001) increases in the percentages of neutrophils, while pretreatment with *M. africana* stembark extract did not affect the increases induced by paracetamol. There were significant (p<0.05-0.001) reductions in the percentages of lymphocytes, monocytes, eosinophils and basophils following paracetamol administration. Further significant (p<0.01) reductions in the number of monocytes and eosinophils were observed in the extract/silymarin–treated rats (Table 1). 

**Evaluation of the effect of** ***M. africana*** **stembark on liver function test of paracetamol-intoxicated rats**

Administration of paracetamol (2 g/kg) to rats caused a significant (p<0.001) elevation of the levels of AST, ALT, ALP, total cholesterol, total and direct bilirubin and decrease in total protein and albumin levels when compared to control. 

**Table1:** Effect of ethanol extract of *Mammea africana* stembark on the hematological parameters of rats with paracetamol -induced hepatotoxicity

**Parameters** **Treatment ** **Dose (m** **g** **/kg)**	**RBC** **(X 10** ^12^ **/l)**	**PCV** **(%)**	**Hb** **(g/dl)**	**WBC** **(X 10** ^9^ **/l)**	**Neutrophils** **(%)**	**Lymphocytes** **(%)**	**Monocytes** **(%)**	**Eosinophils** **(%)**	**Basophils** **(%)**
**Normal control**	3.82 0.32	44.2 10.37	14.80 0.12	11.40 1.85	20.20 1.42	73.8 1.06	2.0 0.05	4.00 0.37	2.20 0.40
**PCM +Dist. water**	3.08 0.34	44.4 1.80	14.82 0.52	10.74 1.80	41.60 1.23^c^	54.4 2.39 c	1.0 0.02 c	2.80 0.96 a	0.22 0.20^c^
**MA. 30mg/kg + PCM**	3.98 0.52	46.2 1.24	15.40 0.34	9.92 1.28	36.91 1.65 c	62.6 2.47 b	1.0 0.01 c	0.00 0.00^c^,^e^	0.00 0.00 c
**MA. 60mg/kg+ PCM**	4.13 0.26	43.5 2.63	14.62 0.63	9.80 1.34	43.22 2.02 c	56.6 2.30 c	0.4 0.04^c^,^f^	0.20 0.20^c^,^e^	0.00 0.00 c
**MA. 90mg/kg+ PCM**	4.18 0.60	44.8 1.15	14.62 0.34	11.36 1.24	35.80 2.66 c	61.2 1.57 b	0.8 0.02^c^,^e^	2.00 0.14 a	0.00 0.00 c
**Silymarin 100mg/kg + PCM**	4.05 0.12	43.2 1.56	14.80 0.12	9.66 2.34	30.43 1.47^b^,^f^	68.2 2.57 e	1.0 0.04 c	0.41 0.40^c^,^e^	0.00 0.00 c

Data were expressed as mean SEM.

a (p<0.05),

b (p<0.01),

c (p<0.001) represent the level of significance when compared to control.

d (p<0.05),

e (p<0.01)

f (p<0.001) represent the level of significance when compared to paracetamol( n = 6).

**Table 2:** Effect of *Mammea africana* stembark on liver functions in paracetamol-intoxicated rats

**Parameters/** **Treatment**	**Total protein** **(g/dl)**	**Albumin** **(g/dl)**	**Total bilirubin** **(µMol/l)**	**Direct** **Bilirubin** **(µMol/l)**	**AST** **(U/L)**	**ALT** **(U/L)**	**ALP** **(U/L)**	**Total cholesterol**
**Normal control**	6.98 ± 0.14	4.25 ± 0.15	5.02 ± 2.81^f^	2.08 ± 2.67	66.42 ± 10.21	26.16 ± 1.77	107.98 ± 6.50	80.86 ± 1.23
**PCM +Dist. water**	3.18 ± 0.42^c^	1.64 ± 0.65^c^	16.88 ± 3.48^c^	10.54 ± 3.08^c^	153.40 ± 18.87^c^	62.18 ± 12.32^c^	180.54 ± 27.56^c^	154.6 ± 3.12^c^
**MA. 30mg/kg + PCM**	4.95 ± 0.44^a^	2.86 ± 0.24^d^	11.62 ± 2.29^e^	7.68 ± 2.08^b^	123.96 ± 8.01^c^^f^	51.88 ± 4.70^c^^f^	165.18 ± 12.94^c^^f^	128.5 ± 2.87^c^^f^
**MA. 60mg/kg+ PCM**	7.13 ± 0.33^e^	3.56 ± 0.28^e^	9.18 ± 1.81^f^	5.18 ± 2.15^f^	92.06 ± 8.34^a^^f^	37.36 ± 8.84^c^^f^	140.08 ± 36.31^f^	88.22 ± 3.42^b^^f^
**MA. 90mg/kg+ PCM**	7.04 ± 0.66^f^	3.94 ± 0.48^f^	5.92 ± 1.31^c^	3.78 ± 1.56^f^	91.74 ± 10.01^f^	36.14 ± 2.58^e^^f^	119.02 ± 12.86^f^	74.43 ± 1.13^f^
**Silymarin 100mg/kg + PCM**	7.16 ± 0.32^f^	4.06 ± 0.25^f^	6.21 ± 1.16^e^	2.26 ± 0.75^f^	95.60 ± 20.56^f^	66.16 ± 10.56^f^	196.28 ± 28.42	78.33 ± 1.96^f^

Data were expressed as mean SEM.

a (p<0.05),

b (p<0.01),

c (p<0.001) represent the level of significance when compared to control.

d (p<0.05),

e (p<0.01)

f (p<0.001) represent the level of significance when compared to paracetamol n = 6).

There were significant (p<0.01 - 0.001) decreases in these enzymes levels and the level of total cholesterol, total and direct bilirubin in the groups pre-administered with the stembark extract of *M. africana *(30–90 mg/kg, groups C to E) when compared with the paracetamol group, which were dose-dependent. Total protein and albumin levels were significantly (p<0.05-0.001) elevated in a dose-dependent manner in the groups pre-treated with the stembark extract when compared to the paracetamol group. The effects of the highest dose of the extract on all of the parameters evaluated were comparable to those of silymarin (Table 2).

**Effect of stembark extract on liver weight**

Liver weights of rats treated with paracetamol were significantly (p<0.001) increased when compared to those of the control group. However, animals in groups pre-treated with the stembark extract and silymarin showed a significant (p<0.01–0.001) decrease in weight when compared to control (Table 3).

**Table 3:** Effect of *Mammea africana* stembark extract on liver weight in paracetamol-intoxicated rats

**Parameters/** **Treatment**	** Liver** **(g)**
**Normal control**	5.45 ±0.53
**PCM +Dist. water**	9.85±0.16^c^
**MA. 30mg/kg + PCM**	6.82±0.24^cd^
**MA. 60mg/kg+ PCM**	6.09±0.65^bf^
**MA. 90mg/kg+ PCM**	5.66± 0.87^cf^
**Silymarin 100mg/kg + PCM**	5.53 ±0.14^cf^

Data were expressed as mean SEM. a (p<0.05), b (p<0.01), c (p<0.001) represent the level of significance when compared to control. d (p<0.05), e (p<0.01) and f (p<0.001) represent the level of significance when compared to paracetamol n = 6).

**Effect of stembark extract on the levels of liver antioxidant enzymes**

Paracetamol treatment caused significant (p<0.001) decreases in the activities of SOD, CAT, GPx and GSH level in liver tissue when compared with control group (Table 4). Pre-treatment with stembark extract of *M. africana* (30 – 90 mg/kg) resulted in a significant (p< 0.05–0.001) increase in the activities of SOD, CAT, GPx and GSH level. Silymarin-treated animals also showed a significant (p< 0.001) increase in antioxidant enzymes (SOD, CAT, and GPx) and GSH levels compared to paracetamol-treated rats (Table 4).

**Table 4:** Effect of *Mammea africana* stembark extract on liver antioxidant enzymes in paracetamol-intoxicated rats.

**Parameters/** **Treatment**	**SOD** **(U/mg of protein)**	**CAT** **(U/mg of protein)**	**GPx** **(U/mg of protein)**	**GSH** **(µg/mg of protein)**
**Normal control**	22.86 ± 0.53	55.68 ± 1.86	26.42 ± 0.66	0.35 ± 0.02
**PCM +Dist. Water**	9.85 ± 0.16^c^	25.40 ± 1.64^c^	10.55 ± 0.42^c^	0.14 ± 0.01^c^
**MA. 30mg/kg + PCM**	12.82 ± 0.24^c^^d^	41.02 ± 1.38^c^^d^	16.04 ± 0.54^c^	0.20 ± 0.01^c^^e^
**MA. 60mg/kg+ PCM**	16.65 ± 0.65^b^^f^	46.15 ± 1.41^f^	22.86 ± 0.25^c^^f^	0.25 ± 0.02^b^^f^
**MA. 90mg/kg+ PCM**	20.09 ± 0.87^c^^f^	51.04 ± 1.22^f^	24.24 ± 1.65^b^^f^	0.29 ± 0.01^a^^f^
**Silymarin 100mg/kg + PCM**	21.66 ± 0.14^c^^f^	53.53 ± 2.12^f^	25.86 ± 1.14^f^	0.33 ± 0.02^f^

Data were expressed as mean SEM.

a (p<0.05),

b (p<0.01),

c (p<0.001) represent the level of significance when compared to control.

d (p<0.05),

e (p<0.01)

f (p<0.001) represent the level of significance when compared to paracetamol (n = 6).

**Histopathological studies of rats liver with paracetamol -induced hepatotoxicity**

**Figures 1:**
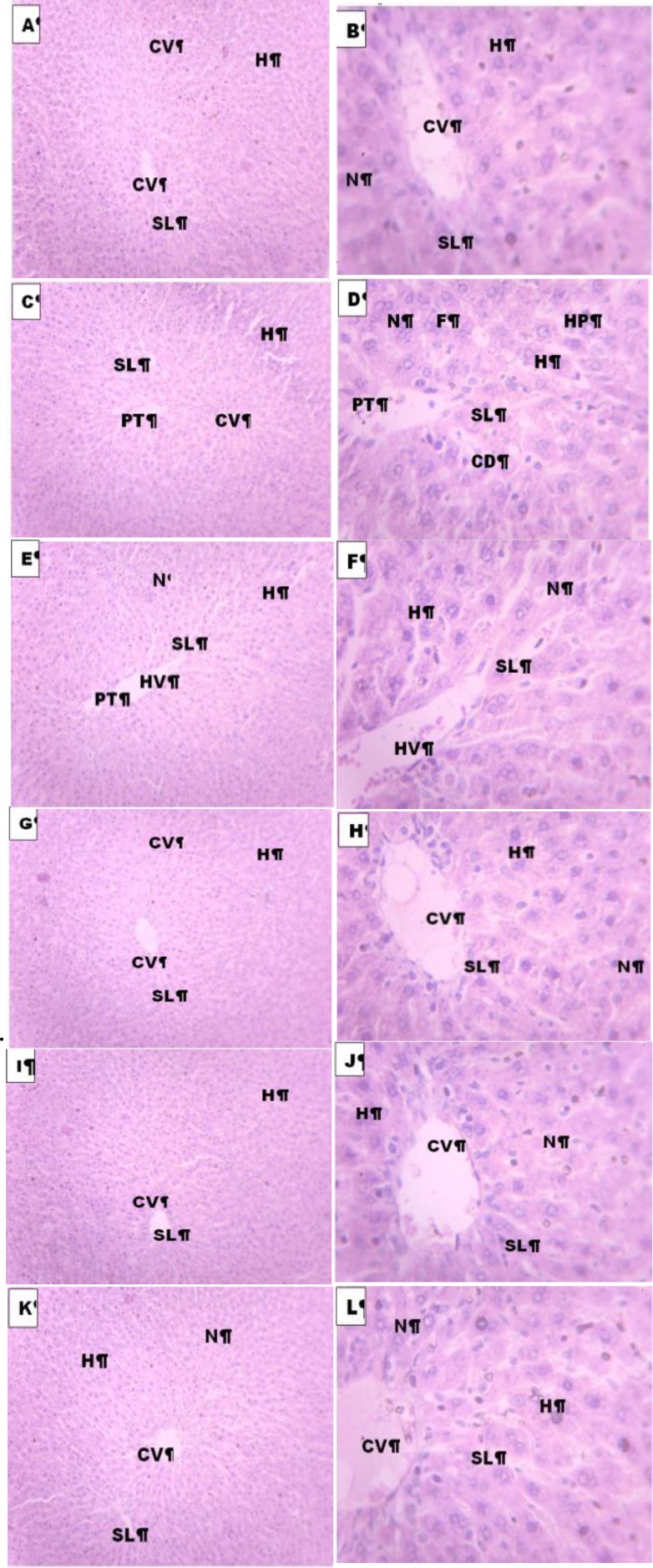
Histological photomicrograph of the liver tissue stained with Heamatoxylin and Eosin techniques of control group (A and B), paracetamol-treated group (C and D), Animals treated with *M. africana *extract, 30 mg/kg (E and E), 60 mg/kg (G and H) 90 mg/kg (I and J) and treated with silymarin group (100mg/kg), (K and L). CV – Central vein, H- Hepatocytes N-Nucleus, RF –Reticular fibers, SL-Sinusoidal lining, PT – portal triad HV- Hepatic vein, HP- Hyperplasia, and CD – Cellular degeneration. Magnification for A, C E, G K and L is X100 but for B, D, F, H, J is X400

Histopathological examination of liver sections of normal control group showed normal cellular architecture with distinct hepatic cells, sinusoidal spaces and central vein (Figures 1 A and B). 

Disarrangement of normal hepatic cells with centrilobular necrosis, hyperplasia, vascular and cellular degeneration, polymorphonuclear aggregation, inflammation and fatty degeneration were observed in paracetamol-treated rats of group B (Figures 1 C and D).

The liver sections of the rats treated with stembark extract of* M. africana *(30 - 90 mg/kg) of groups C, D and E showed signs of protection evidenced by the reduction/ absence of inflammatory cells, vascular congestion and degeneration, cellular degeneration, necrosis and vacuoles (Figure 1 C - J), while the liver sections of the rats treated with silymarin (100 mg/kg) in group F showed significant reduction in fatty degeneration and absence of necrosis and inflammation (Figures 1 K and L).

## Discussion

In this study, paracetamol administration was found to cause elevation of enzymes levels such as AST, ALT, ALP, total cholesterol, total and direct bilirubin and decrease total protein

and albumin levels. These conditions were however reversed by pre-treatment with the extract and standard drug, silymarin. Paracetamol is a useful experimental model for evaluation of hepatoprotective activity of medicinal plants and drug (**Gite et al., 2010**). 

Paracetamol is reported to produce acute toxic effect at high doses which leads to liver damage as a result of its bioactivation by the cytochrome P450 system to a toxic electrophile, *N*-acetyl *p*-benzoquinone imine (NAPQI), which covalently binds to tissue macromolecules, probably oxidizes lipids or the critical sulfhydryl groups (protein thiols) and alters the homeostasis of calcium (Davern et al., 2006 ▶; Das and Sarma, 2009 ▶; Marotta et al., 2009 ▶; Jaeschkea and Bajt, 2010 ▶). This could have been the case in this study. 

The massive production of reactive species may lead to depletion of physiological protective moieties (glutathione, α-tocopherol, etc.), ensuing wide-spread propagation of the alkylation and peroxidation, and damaging the macromolecules in vital biomembranes (Gilani et al., 2005 ▶). Induction of cytochrome and depletion of glutathione are the major predisposing factors to liver injury (Gupta et al., 2006 ▶). 

Damage to the structural integrity of liver is reflected by an increase in the levels of serum transaminases AST, ALT and ALP, because they are cytoplasmic in location and are released into the circulation after cellular damage. Assessment of liver function can be undertaken by estimating the activities of serum ALT, AST, ALP, bilirubin (total and direct), total cholesterol, total protein and albumin which are originally present in the cytoplasm (Manokaran et al., 2008 ▶). When there is hepatopathy, these enzymes and molecules leak into the blood stream serving as an indicator of the liver damage (Nkosi et al., 2005 ▶). The aminotransferases (ALT and AST) are the most commonly used specific indicators of hepatic necrosis (Dama et al., 2011). ALT is a sensitive indicator of acute liver damage and elevation of this enzyme in non-hepatic diseases is unusual. ALT is more selectively a liver paranchymal enzyme than AST (Nkosi et al., 2005 ▶).

 Abnormally high levels of serum ALT, AST, ALP, total bilirubin (total and direct), and total cholesterol and decreases in total protein and albumin levels observed in paracetamol group in our study are the consequence of paracetamol-induced liver dysfunction and denote the damage to the hepatic cells due to paracetamol intoxication (Parmar et al., 2010 ▶; Mandade, 2011 ▶).

Serum ALP, bilirubin and total protein levels are related to the function of hepatic cell. Elevated level of ALP suggests biliary damage or an obstruction of the biliary tree, which disrupts the flow of blood to the liver (Kouitchev et al., 2007 ▶; Farida et al., 2012 ▶). The reversal of increased serum enzymes in paracetamol-induced liver damage by the extract may be due to the prevention of the leakage of intracellular enzymes by its membrane stabilizing activity. 

In the present study, reduction in serum total protein and albumin levels were observed in paracetamol-treated rats which may be associated with the decrease in the number of hepatocytes which in turn may result in a decrease in hepatic capacity to synthesize protein and albumin. Hypoalbuminemia portrays an advanced chronic liver diseases and a reduction in serum total protein level is a useful index of the severity of cellular dysfunction in chronic liver diseases. Decreased levels of total protein and albumin as recorded in paracetamol-treated rats revealed the severity of hepatopathy and may be attributed to the damage produced and localized in the endoplasmic reticulum leading to decrease in protein synthesis (Kanchana and Sadiq, 2010 ▶). This negative effect on total protein and albumin was reversed in extract-pretreated groups and is clear indication of the improvement of the functional status of the liver cells by the extract.

Bilirubin, a metabolic product of hemoglobin, undergoes conjugation with glucuronic acid in hepatocytes to increase its water solubility. Elevated levels of bilirubin due to paracetamol intoxication in this study may be attributed to excessive heme destruction and blockage of biliary tract resulting in inhibition of the conjugation reaction and release of unconjugated bilirubin from damaged hepatocytes (Ali et al., 2010 ▶). Decrease in serum bilirubin after treatment with the extract in liver with paracetamol-induced damage, indicated the effectiveness of the extract to restore normal functional status of the liver.

Hepatotoxic substances are known to cause impairment of cholesterol metabolism leading to an increase in serum levels of cholesterol causing fatty liver (Farida et al., 2012 ▶). Paracetamol-induced toxicity in rats may have altered membrane structure and function as well as lipids metabolism in the liver as suggested by increased cholesterol levels in rats. This result is an indication of membrane rigidity caused by paracetamol. This effect was reduced by the protective activity of the stembark extract which restored the level of total cholesterol to near normal.

Superoxide dismutase, one of the most important intracellular antioxidant enzymes present in all aerobic cells, has an anti-toxic effect against superoxide H anion (Loki and Rajamohan, 2003 ▶). It scavenges the superoxide anion to form hydrogen peroxide, hence reducing the toxic effects caused by these radicals. In this study, it was observed that *M. africana* stembark extract significantly increased hepatic SOD activity in paracetamol-induced liver damage in rats. This showed that *M. africana* can reduce reactive free radicals, thereby reducing oxidative damage to the tissues besides improving activity of hepatic antioxidant enzymes.

Catalase is a haemoprotein that protects cells from the accumulation of H_2_O_2_ by reducing it to form H_2_O and O_2_ or by using it as an oxidant in which it works as peroxidase. Therefore, decreased CAT activity may result in a number of deleterious effects due to the inability to scavenge superoxide radical and hydrogen peroxide. However, the activities of SOD and CAT in the liver were significantly reduced in paracetamol-treated rats than the normal rats. This effect suggests excessive formation of free radicals and activation of lipid peroxidation system resulting in tissue damages (Kuriakose and Kurup, 2008 ▶). Doses of the stembark extract (30 - 90 mg/kg) increases the level of CAT in a similar manner as silymarin, the standard hepatoprotective drug, revealing its potentials to prevent the accumulation of excessive free radicals, thus protecting the liver from paracetamol intoxication.

Glutathione (GSH) is one of the tripeptide and nonenzymatic biological antioxidants that is present in high quantity in the liver. It helps to remove free radical species such as hydrogen peroxide, superoxide radicals, alkoxy radicals and maintain membrane protein thiols, through GPx and GST activities and maintenance of membrane protein thiols (Prakash et al., 2001 ▶; Mandade, 2011 ▶). Determination of total GSH is a key factor to show the antioxidant reserve in the organism (Odukoya et al, 2007 ▶; Balouchxadehet al 2011 ▶). Excessive peroxidation causes increased GSH consumption. GSH is a scavenger of toxic metabolites, including NAPQI, which is a metabolite of APAP (Hwang et al., 2008 ▶; Yuan et al., 2010 ▶). Reduced level of GSH is implicated in the enhancement of lipid peroxidation in paracetamol-treated rats (Parmer et al., 2010 ▶). Administration of *M. africana *and silymarin significantly increased the level of GPx and GSH in a dose-dependent manner portraying its ability to scavenge these free radicals. The activities of the extract and silymarin is in agreement with a number of previous studies on hepatoprotective effect of plant extract and silymarin (Oyegbami and Odetola, 2010 ▶; Zamani-Moghaddani et al., 2012 ▶). 

Silymarin, a mixture of flavonolignans consisting of silybin, silychristin, silydianin and isosilybin, is a standardized seed extract of *Silybum marianum* (Madani et al, 2008 ▶) .Studies in cell culture and animal models clearly show its hepatoprotective property against carbon tetrachloride, paracetamol, Amanita phalloide toxin and thioacetamide (Muriel and Mourelle, 1990 ▶; Muriel et al., 1992 ▶; Das, 2012 ▶). Silymarin at doses up to 100 mg/kg has been used as a standard hepatoprotective agent by numerous investigators and it exerts hepatoprotective effect due to its antioxidant and scavenging properties (Kosina et al., 2002 ▶).

Certain drugs including alkylating cytotoxic agents could also affect blood formation rate and hematological parameters (Adeneye et al., 2008 ▶). Administration of paracetamol to rats increases erythrocyte membrane peroxidation, which may also lead to haemolytic changes. Treatment of rats with paracetamol in this study, did not significantly affect the haematological parameters such as RBC, Hb, PCV, and WBC levels except increases in neutrophils percentage and reductions in the percentages of monocytes, eosinophils and basophils. Pre-administration of *M. africana* extract to these animals also did not affect the above states though further reductions were observed in the percentages of monocytes and eosinophils as compared to the paracetamol-treated group. The histological findings corroborate that of the biochemical results as the degree of injury induced by paracetamol was reduced significantly by pretreatment with the stembark extract.

The stembark extract and fractions have been reported to exhibit strong cellular antioxidant activity in whole blood, neutrophils (extracellular and intracellular) and macrophages (Okokon et al., 2013). Similarly, the cytotoxic coumarins isolated from the stembark have also been reported to exert strong antioxidant activity (Nguelefack-Mbuyo et al., 2010 ▶). This activity demonstrates the potential of the extract to inhibit reactive oxygen species (ROS) and scavenge free radicals like superoxide, hydrogen peroxide, etc. which can be attributed to the presence of coumarins and other phenolic compounds in the stembark what were reported earlier (Carpenter et al 1971 ▶; Games, 1972 ▶; Crichton and Waterman, 1978 ▶; Ouahouo et al., 2004 ▶; Nguelefack-Mbuyo et al., 2010 ▶; Okokon et al., 2013). The strong antioxidant activity of this extract explains the significant hepatoprotective activity of the stembark extract. Free radical mediated process has been associated with pathogenesis of most of the diseases. The activities of antioxidant counteract the redox state precipitated intracellularly and hence ensure hepatoprotection against paracetamol-induced liver injury. The antioxidant activity of this extract may as well explain the mechanism of action of the observed hepatoprotective activity of *M. africana* which may be related to inhibition of lipid peroxidation and enhancement of antioxidant enzyme levels in addition to free radicals scavenging action.

The study shows that *Mammea africana* possesses strong hepatoprotective activity which is due to its antioxidant activity precipitated by its chemical constituents. This confirms the use of *M. africana* stembark as an antidote in traditional medicine.
